# Improvements in Injection Moulds Cooling and Manufacturing Efficiency Achieved by Wire Arc Additive Manufacturing Using Conformal Cooling Concept

**DOI:** 10.3390/polym16213057

**Published:** 2024-10-30

**Authors:** Alejandro Marqués, Jose Antonio Dieste, Iván Monzón, Alberto Laguía, Pascual Gracia, Carlos Javierre, Isabel Clavería, Daniel Elduque

**Affiliations:** 1 R&D Department, Aitiip Fundation, Empresarium Industrial Site, C/Romero, No. 12, 50720 Zaragoza, Spain; joseantonio.dieste@aitiip.com (J.A.D.); ivan.monzon@aitiip.com (I.M.); alberto.laguia@aitiip.com (A.L.); pascual.gracia@aitiip.com (P.G.); 2 Department of Mechanical Engineering, University of Zaragoza, C/María de Luna, 50720 Zaragoza, Spain; carlos.javierre@unizar.es (C.J.); iclaver@unizar.es (I.C.); delduque@unizar.es (D.E.)

**Keywords:** additive manufacturing, big area additive manufacturing, injection moulding, wire arc additive manufacturing, conformal cooling

## Abstract

The plastic injection moulding industry is a constantly developing industrial field. This industrial process requires the manufacturing of metal moulds using complex heating and cooling systems. The purpose of this research is to optimize both the plastic injection moulding process and the mould manufacturing process itself by combining practices in this industry with current additive manufacturing technologies, specifically Wire Arc Additive Manufacturing (WAAM) technology. A mould punch was manufactured by using both WAAM technology, whose internal cooling system has been designed under the concept of Conformal Cooling, and conventional cooling channel designs and manufacturing techniques in order to carry out a comparative analysis. Theoretical results obtained by CAE methods showed an improvement in heat extraction in the WAAM mould. In addition, the WAAM mould was able to achieve better temperature homogeneity in the final part, minimizing deformations in the final part after extraction. Finally, the WAAM manufacturing process was proven to be more efficient in terms of material consumption than the conventional mould, reducing the buy-to-fly ratio of the part by 5.11.

## 1. Introduction

Today’s industry trends demand large batches of identical parts in a short time and at minimal cost. This situation has led to the development of the injection moulding industry. This industry has not stopped growing year by year, with an estimated market size of $261.8 billion in 2021, and an estimated growth of 4.8% by 2030 according to the Grand View Research database [1].

Plastic injection moulding leads the market due to its capacity to produce large batches of parts, as mentioned, as well as the ability of this process to maintain high standards of precision, its customization capabilities, and the outstanding final quality this technology offers [2]. Moreover, thanks to the high optimization of this process [3,4,5], plastic has replaced other conventional materials such as metal, ceramic, or glass in certain industries [6,7].

Plastics Europe reports that, out of all the types of plastics available, the thermoplastic variations [8], namely polypropylene (PP), low-density polyethylene (LDPE), and high-density polyethylene (HDPE), are the most sought after. HDPE, in particular, accounts for 12% of the overall plastic demand in Europe [9]. This polymer is enormously versatile, and it is suited to a wide range of applications. The producer or converter can modify the impact and tear resistance, transparency, tactility, flexibility, formability, and the coating/laminating/printing capability of polyethylene by adjusting its formulation and thickness [10]. HDPE can be recycled, and many products, including bin bags, agricultural films, park benches, bollards, and waste bins, use recycled polyethylene [11,12]. Moreover, due to its high calorific value, this thermoplastic is also an excellent candidate for energy recovery through clean incineration [13,14]. These properties make HDPE quite a common material in various industries, such as packaging [15], construction [16,17], agriculture [18,19], healthcare [20,21] and automobility [22].

Focusing on the injection moulding process, it can be broken down into five stages: mould filling, packing, cooling, plasticizing, and ejection of the finished product [23]. Regarding mould filling, this process is conducted in toolings in which, by means of temperature and pressure, the geometry of the part is formed, the so-called moulds, consisting of two parts, the punch and the cavity. One of the key issues in this process is the design and optimization of the heating and cooling channels for the injected material, with the cooling process being the most influential in terms of total cycle time, representing between 50% and 70% of it [24]. In addition, throughout the cooling cycle of the injected part, the temperature distribution in the mould and its cooling rate are critical for the quality of the injected part. Insufficient or non-uniform cooling can affect the part geometry, shrinking or deforming it [25,26]. In addition, a non-uniform temperature distribution may also cause undesirable effects on the part, which can be observed in its surface finish (such as poor surface finish areas or the appearance of shiny spots) [27].

Conventional cooling methods, based on drilling or the use of bubblers, baffles, and isobars on the mould, are producible by conventional manufacturing methods. This procedure is suitable for simple final part geometries; however, it is not appropriate when the part to be injected is geometrically complex [28]. With an emphasis on the cooling cycle of the final parts, the concept of Conformal Cooling (CC) was developed. Based on this concept, the cooling channels of the moulds are designed to follow the shape of the final part [29,30,31,32], seeking to minimize the distance between the cooling source and the plastic injected part. This optimization results in enhanced heat transfer with the final part, which in turn improves cycle time [33,34,35]. In addition, several studies have shown significant improvements in heat distribution across the tooling [36,37], a reduction in the process temperature obtained through channel optimization [38], and a reduction in final part deformation [39]. However, in larger moulds, the manufacturing of these channels is usually complex due to the limitations of the machining processes themselves [40]. For this reason, a mould whose cooling system is based on the CC concept requires, in most instances, advanced manufacturing methods. As common manufacturing methods only allow for straight holes to be drilled, adapting the channels to the shape of complex geometries requires intricate successions of straight holes, which demands multiple secondary operations, like plugging the channels, polishing the plug area, etc. Another method of fabrication would be to design inserts for the areas of the channels where their geometry does not allow straight holes to be drilled. These inserts could consist of several pieces, separately machined and installed in the indicated area of the mould. This method has the disadvantage of adding secondary parts to the mould, which must be adjusted after installation, making the system even more complex. For both cases, an added problem is the increased possibility of leaks, as the number of areas requiring a perfect seal increases. For these reasons, Conformal Cooling designed channels generally cannot be achieved by conventional machining methods, making it necessary to resort to technologies such as additive manufacturing (AM) [41]. It should be noted that the CC concept is applicable to any manufacturing process involving a thermal cycle of heating and cooling by means of fluid, such as injection moulding (as in the case of the current study), thermoforming, press-forming, etc.

The industrial use of additive manufacturing has become increasingly important, and it has already demonstrated its full applicability in the plastic injection moulding field [42,43,44]. AM is being utilized to manufacture efficiently functioning metallic parts, and its use is becoming more widespread in several industries. This technology is highly researched due to its ability to produce complex geometries while maintaining high mechanical properties [45,46,47]. In addition, it has the potential to save both materials and energy compared to traditional manufacturing methods [48,49,50]. One notable aspect of AM is the procedure known as BAAM (Big Area Additive Manufacturing), which is a dimensional specification of AM processes. BAAM includes additive manufacturing machines that can create larger dimensions than standard 3D printers (400 × 400 × 200 mm) and produce printed volumes of several cubic meters [51,52,53].

AM is a widely used manufacturing process in several industries, and it has the advantage of working with different materials, among other benefits. Focusing on the plastic injection moulding process, due to the high mechanical and thermal requirements of the moulds, as well as their need for manufacturing large parts, the possibilities for AM in terms of filler materials are restricted to the use of metallic materials. This technology is known as Metallic Additive Manufacturing (MAM) [54,55]. Regarding MAM technologies, depending on the filler material used, we can name two main groups: powder-feed technologies and wire-feed technologies [56,57]. For the specific case of large toolings, the printing area limitations of powder-feed technologies (limited to maximum dimensions of 300 × 300 mm for the printing bed, with part heights of less than 500 mm) make them unfeasible.

On the other hand, wire-feed technologies are capable of manufacturing large metal parts by the superposition of welding layers [58]. This process can be performed by a collaborative robot adapted to the additive process, eliminating the size limitation of the printing base [59]. Wire Arc Additive Manufacturing (WAAM) is the most representative technology within this group. It can be defined as a manufacturing process which involves the use of an electric arc to melt the apported electrode, creating layers with the deposited material. In this study, the chosen WAAM method has been Gas Metal Arc Welding (GMAW) [60]. In this welding process, an electric arc is generated between a consumable electrode and the workpiece, generating high temperatures that melt the upper surface layers of the base metal and the tip of the electrode. At this point, the electrode molten metal is deposited onto the upper molten surface layer of the workpiece, creating a unique metallic part. An inert gas-protected atmosphere, wire feed, and welding current are the main inputs required by the system [61,62]. This process presents several advantages, such as a high deposition rate, low equipment costs, and a small amount of residues. On the other hand, it should be noted that this process is relatively young and is therefore not developed to the same level as conventional manufacturing systems. In addition, another sensitive point to consider is the limited quality of the printed surfaces, making a post-processing of the surfaces with precision finishing requirements indispensable [63,64]. The consequence of these circumstances is the need to design a specific CAD (computer-aided design) for AM, removing certain features such as threads or small holes that should be manufactured in the second-stage post-processing. In addition, due to the necessity of removing material to achieve a high-quality final part, this AM specific design must have a design that includes over-thickness on the outer surface, which will then be machined [65,66,67]. This over-thickness also serves as a safety element in the event of a failure of the printing process. Another circumstance that must be considered during the design stage is the process temperature of the WAAM system. During this additive process, the material reaches temperatures of up to 450 °C, expanding the geometry. To compensate for this expansion, the part must be designed taking into account this shrinkage, so that the cold tooling accomplishes the target geometry. This specific CAD has been named WAAM-process-adapted CAD.

Based on the information presented, there is a lack of literature regarding large-size tooling designed according to the CC concept. The use of Metal Additive Manufacturing is shown to be the most suitable for the fabrication of these tools. However, the scale restrictions imposed by contemporary technologies have prevented this phenomenon from being fully developed. Therefore, the objective of this paper will be the study of a large punch mould manufactured by WAAM technology, designed according to the CC concept. The manufacturing parameters of this mould, as well as the performance of its cooling system, will be compared with those of an identical theoretical mould, with a conventional cooling system, machined by conventional manufacturing techniques.

## 2. Materials and Methods

**Figure 1:**
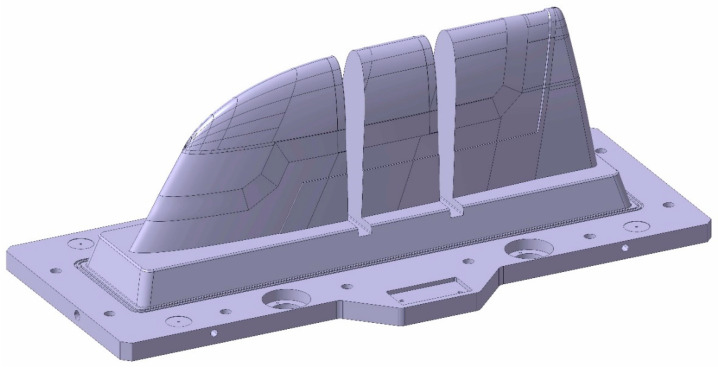
Final mould design CAD.

As introduced, the aim of this article is the manufacturing comparison of two similar large-dimension moulds (Figure 1), with sizes of approximately 820 × 1510 × 512 mm, in terms of manufacturing parameters and cooling performance.

The first mould is based on the concept of Conformal Cooling, for which cooling channels adapted to the shape of the final part have been designed. This mould has been manufactured by Metal Additive Manufacturing, specifically by WAAM technology, due to the complexity of the design, which is not achievable by conventional manufacturing methods. This mould is henceforth named the WAAM mould for easy identification. On the other hand, the second mould, which will be called the Conventional mould, was used as a reference to compare with the WAAM mould. This Conventional mould was designed to be completely manufacturable by conventional manufacturing processes; therefore, its cooling channels were simplified, moving away from the concept of Conformal Cooling.

First, a comparison was made at the design level, emphasizing the differences between the two moulds derived from the manufacturing point of view.

Based on the initial design analysis, a comparison was made between the manufacturing processes of the Conventional mould and the WAAM mould. For this purpose, emphasis was placed on the design differences depending on the manufacturing process, as well as on the material consumptions in both processes.

It should be mentioned that the total mould assembly has two parts: punch and cavity. The study is based on the design and manufacturing of the mould punch, as it is the most suitable to be manufactured by additive manufacturing methods; therefore, we always refer to this part of the mould in this article. Regarding the mould cavity, its design and manufacturing were carried out following conventional manufacturing criteria. This decision was based on two main reasons:

- The main concept to be analyzed during this manufacturing study was the ability of the WAAM system to manufacture internal channels according to the Conformal Cooling concept. In the case of the cavity mould, this type of design strategy could be achieved by conventional manufacturing methods with few geometrical constraints and therefore was not of interest for the study.
- During the study, only one WAAM manufacturing system was available. Consequently, the simultaneous fabrication of both parts of the mould was not possible and would have taken excessive manufacturing time.

**Figure 2:**
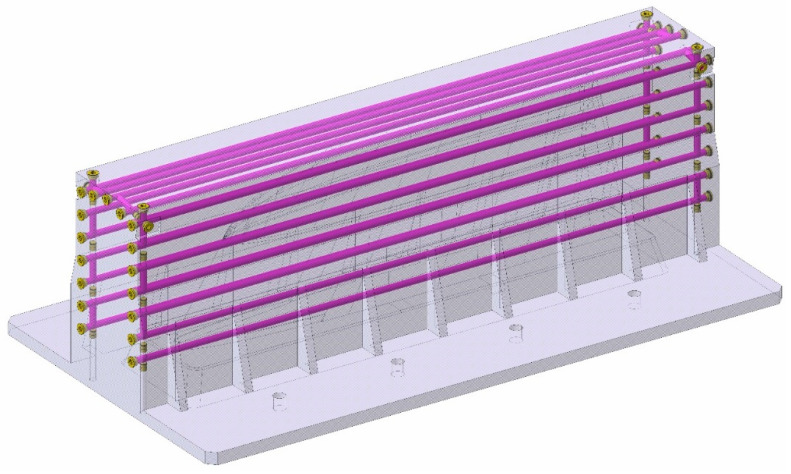
Cavity mould CAD. Cooling channels are highlighted in purple.

The design of the cavity mould is shown in Figure 2, with its internal circuitry highlighted in violet. The Channel diameter is 15 mm.

It should be noted that, along with the study, the displayed images of the mould only include the figure’s footprints parts (punch and cavity), leaving out the casting elements of the system for a better visualization of the mould’ s characteristics.

Regarding the WAAM mould manufacturing process, two different steps should be differentiated: the WAAM process and the second-stage post-processing.

**Figure 3:**
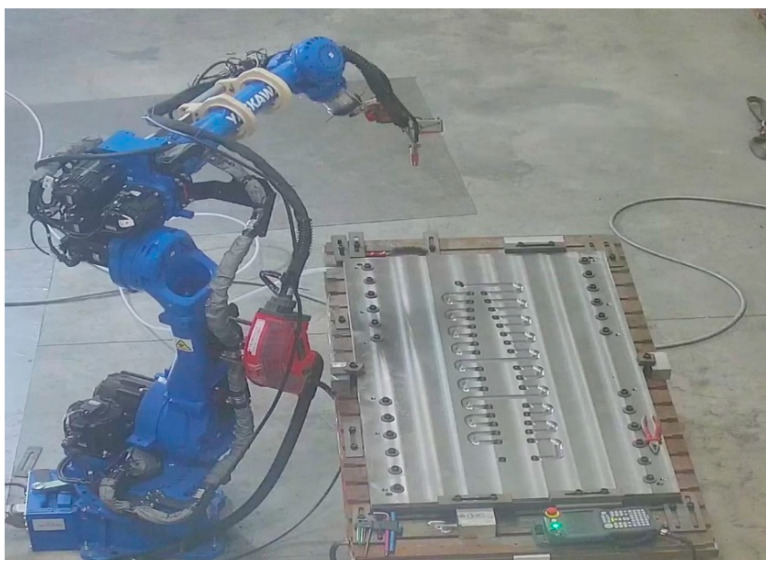
WAAM manufacturing system with mould base Photograph taken at the facilities of Fundación Aitiip.

**Figure 4:**
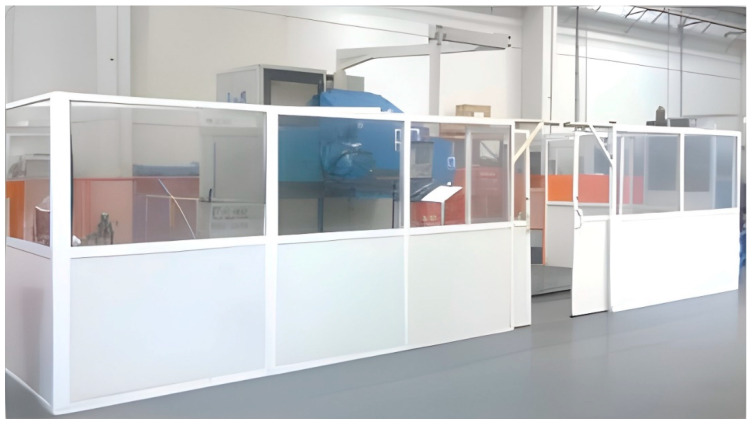
The 5-Axis NC machine, CME FCM 400. Photograph taken at the facilities of Fundación Aitiip. (Reprinted from ref. [50]).

1. The WAAM process was performed by the welding system Fronius 400i [68]. This device was assembled over a collaborative robotic arm Yaskawa GP50 [69], allowing 6 degrees of freedom in the robot’s welding header (Figure 3). This welding system has been tested in the literature previously [70], as well as on multiple occasions at Aitia’s facilities. In terms of software, the movements of the robot are programmed directly from a CAM software (Hyper MILL CAD/CAM 2023) [71], which automatically guides the robot’s path. The resulting movements are post-processed into ISO G-Code language. The resulting file is then simulated in an Off-line Programming Software (Ultimaker Cura 2023) [72] to prevent collisions and ensure that all the movements are within the robot’s range.
2. The post-processing process is the machining process needed to achieve the final dimensional requirements, as well as to perform specific features such as drills, threads, etc. In our specific case, it was carried out by a CME FCM400 5 axis machining center, as shown in Figure 4.

Finally, the cooling cycles proposed for both moulds were simulated. This simulation compared the effect of the designed internal channels on mould cooling cycle performance in both moulds.

**Figure 5:**
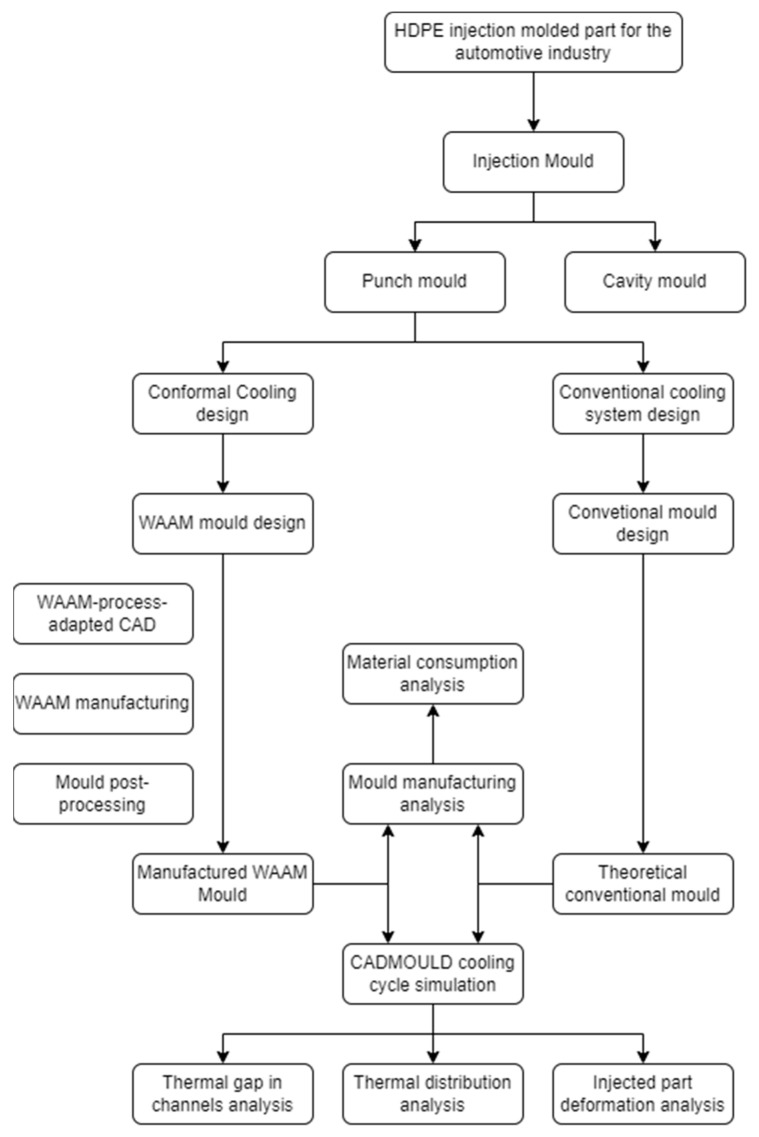
Schematic diagram of the study methodology.

The above-described methodology will be used for the study of the fabrication and performance of a punch mould designed according to the CC concept. This large-size tooling has been manufactured by the WAAM process in order to achieve the design specifications, constituting a unique specimen in terms of size and complexity for metallic additive manufacturing. The methodology of the study is based on the diagram shown in Figure 5, and its applications can be found in Section 3. It shows the comparative analyses carried out between the two moulds, as well as the previous steps carried out.

## 3. Case Study

In this study, an automotive mould designed for the manufacture of injection-moulded HDPE parts was analyzed. From this premise, the concept of Conformal Cooling was developed for the WAAM mould. In order to compare the cooling process, the Conventional mould was designed. The cooling circuits of the Conventional mould were adapted, as far as possible, to the difficult part geometry, while complying with the limitations of conventional manufacturing methods. For its design, a baffle system was used to create the cooling circuits.

To get a broader perspective of the differences between the two toolings, as mentioned above, a comparison of the material consumption across the manufacturing process of both moulds was performed. The WAAM process material consumption data was based on the welding wire consumption, whose specific parameter is called Wire Feed Rate (WFR). This is a direct input of the welding device used. On the other hand, the material consumption in the Conventional mould was calculated according to the tooling machining requirements and based on tooling design experience.

**Figure 6:**
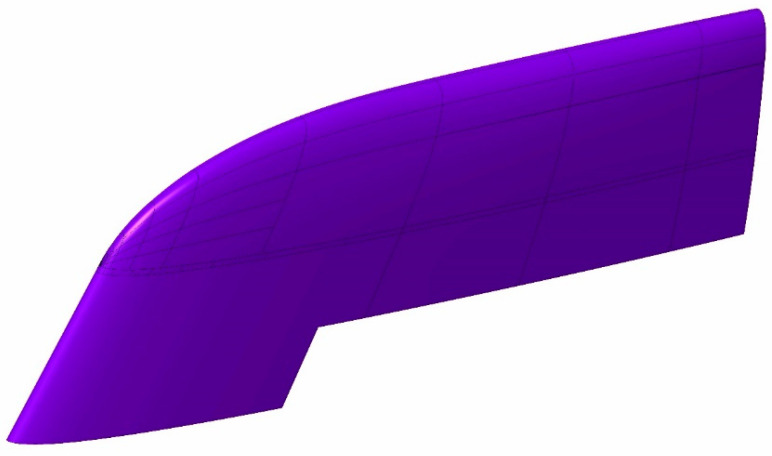
Automotive injection-moulded part CAD.

As previously stated, the final part to be injected was an exterior trim for the automotive industry (Figure 6). As it is a part that does not have high mechanical requirements, the injection material is high-density polyethylene, which is commonly used in the automotive industry [73].

For this study, a thermal and deformation analysis of the final part was performed considering the effect of the internal channels designed and manufactured for the WAAM mould. To this effect, the performance of the CC concept-designed channels was compared with that of the Conventional mould previously presented. The outer geometry of these toolings was identical; however, the distance between the internal channels and the outer surface of the tooling varied considerably. The cooling cycle analyses were carried out by means of the CADMOULD V16 [74] software. CADMOULD is a professional plastic injection moulding simulation software fully integrated in the industry and used in various research articles related to plastic injection moulding [75].

### 3.1. Mould Design Differences

**Figure 7:**
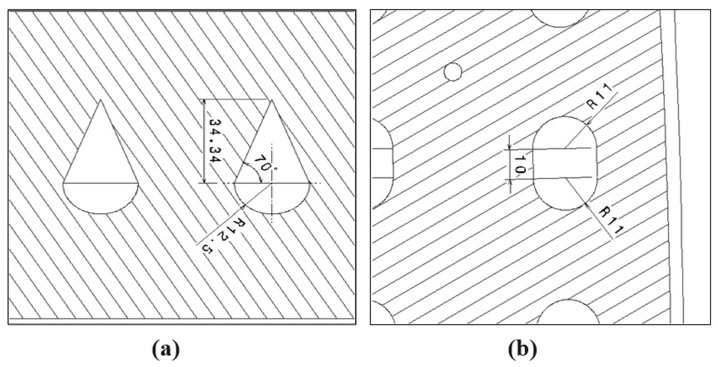
Dimensioned mould drawing. (**a**) Horizontal channel section detail and (**b**) Vertical channel section detail.

Regarding the WAAM mould, for the design and fabrication of the channels, two different design strategies were differentiated, depending on the printing area. The design premise was to maintain the channel section constant throughout the mould, an objective that was considered to be achieved by maintaining section differences of approximately 10% throughout the tooling. This difference is in response to the slight collapse of the horizontal channel geometry in its upper zone, as shown in Figure 7a. This collapse decreases the area of the channel by approximately 10%, according to the tests carried out. For this reason, the vertical channels were designed with a 10% reduction in this area to maintain a constant area along the internal channels. For horizontal channels, whose first circular half is machined on the flat plate on which the impression is made, the upper part of the channel must be closed. For this manufacturing operation, the cantilever angle printed for closing such a channel should be at 20° maximum. This value for the maximum cantilever angle has been obtained based on both own experiments and the existing literature [76,77]. It should be noted that this is a conservative value, aiming to minimize possible collapse errors during construction. As for the channels growing vertically, a geometry easily adaptable to the lower channels was chosen. It should be noted that the cantilever angle of these “vertical” channels is between 5° and 8°. Both channels’ geometries are visualized with their respective dimensions in Figure 7. As for the design cross-section, the channel geometry manages to maintain a reasonably constant cross-section of 0.092 m^2^ in the horizontal channels and 0.098 m^2^ in the vertical channels.

**Figure 8:**
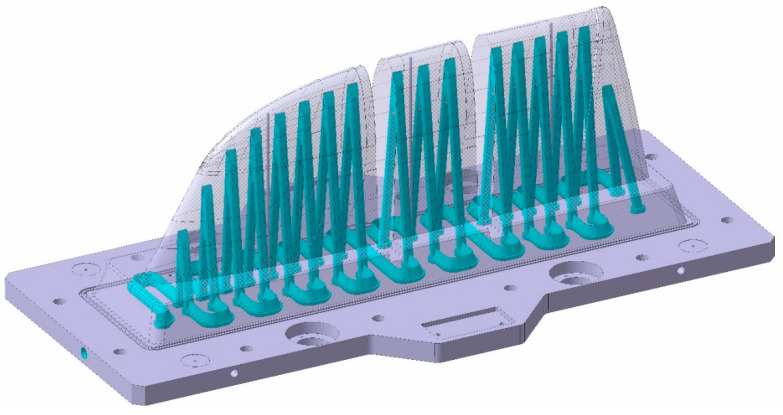
Conformal tooling concept WAAM mould. Inner cooling channels highlighted in blue.

As explained before, the WAAM process is a near- to-shape process. The WAAM fabrication process involves the overlapping of weld seams, which generates irregular side surfaces on the outer surfaces of the geometry. To prevent these irregularities from affecting the final shape of the part [78,79], a specific WAAM-process-adapted CAD was designed, adding over-thickness for safety on the external surfaces to be machined and eliminating elements that were machined later, such as threaded holes, housings, etc. Regarding the inner channels, they have their own printing and design specifications. Inner channels should accomplish a minimum 25 mm security distance from the outer printing surface. Concerning the channels themselves, it is recommended to increase their design dimensions with a theoretical offset of 7.5 mm. The reason is that these areas are heat accumulation zones during the additive process; thus, the material flows more during the welding process. This issue creates wider than normal beads in these print areas. The WAAM tooling cooling channel design is displayed at Figure 8.

**Figure 9:**
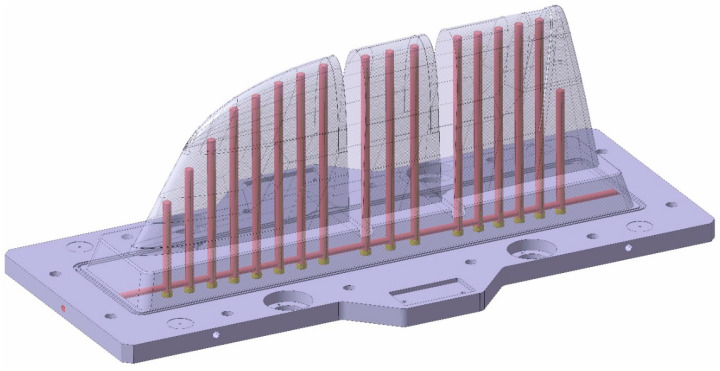
Conventional mould CAD design. Inner cooling channels highlighted in orange.

The so-called Conventional mould was based on the mentioned baffle heating and cooling system (Figure 9). The baffles are a dividing element capable of creating two circuits from a single hole. Thanks to this element, a cooling system is achieved whose channels coincide in the horizontal plane with the channels designed under the concept of Conformal Cooling; however, they are not adapted to the geometry of the final part but positioned in the central area of the mould.

Regarding the complexity of the manufacturing of both moulds, it should be noted that the WAAM process requires only two phases: the additive manufacturing phase and the second-step machining phase. In this sense, the manufacturing process is significantly simplified with respect to the manufacturing of the Conventional mould, since, in order to for it to have the required cooling channels, it would be necessary to drill deep boreholes, which require specific machinery for their execution, being difficult to perform on a technical level [80]. In addition, the Conventional mould requires a system of baffles and plugs, which make up the thermal circuit. The installation of these peripherals greatly increases the complexity of assembly compared to the WAAM mould.

**Figure 10:**
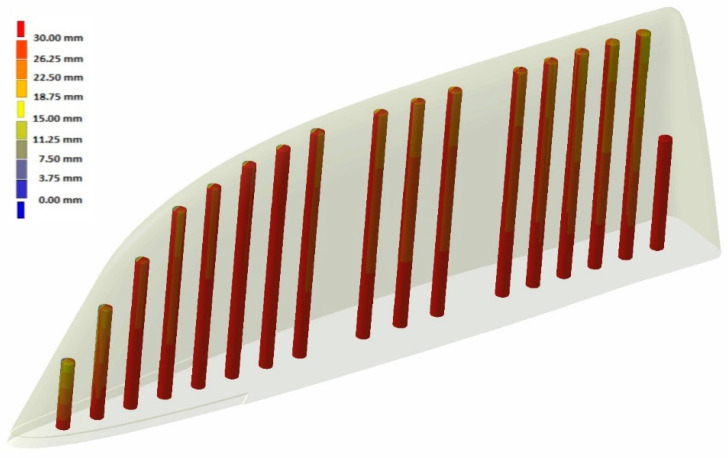
Conventional mould: distance between cooling channels and outer surface.

**Figure 11:**
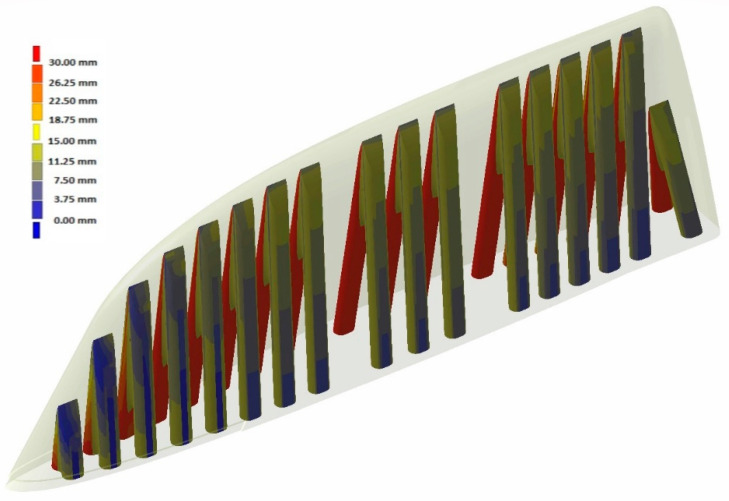
WAAM mould: distance between cooling channels and outer surface.

With the aim of testing the distance of the cooling channels to the workpiece injection surface, a distance analysis was performed. As can be observed, in the case of the Conventional mould (Figure 10 and Figure 11), the cooling channels were mostly at separation distances from the outer surface of about 30 mm. However, the WAAM mould (Figure 11) achieved much smaller clearances, reaching distances close to 5 mm in its best-optimized areas. It should be noted that only the injection area of the tooling has been included in these clearance graphs.

### 3.2. Material Comsumption Description

#### 3.2.1. Conventional Mould Manufacturing

**Figure 12:**
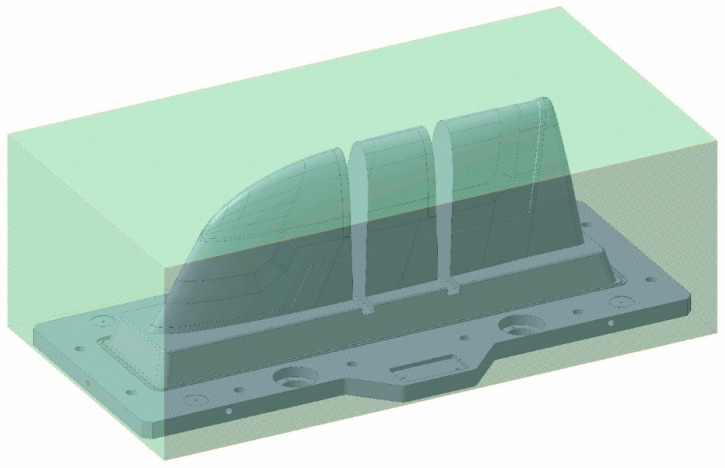
Conventional mould design integrated in its raw steel block.

For the concept of the Conventional mould, the design premise involved making the entire punch mould in one piece. Therefore, the selected raw steel block should accomplish nominal dimensions, plus 5 mm of extra growth. This extra growth was selected according to machining experience, being the minimum growth required to ensure the dimensions of the final mould. The final dimensions of the raw steel block were 825 × 1515 × 517 mm, and its density was 7860 kg/m^3^. Figure 12 displays the Conventional mould design integrated in its raw steel block.

#### 3.2.2. WAAM Mould Manufacturing

**Figure 13:**
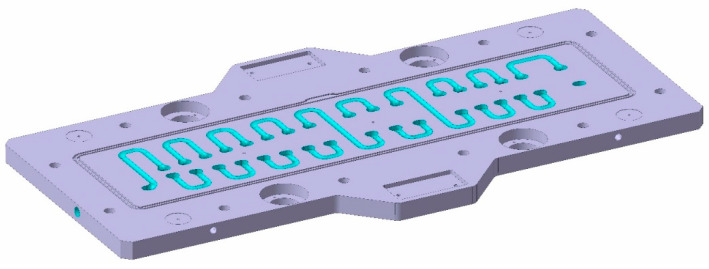
Machined mould base CAD. Manufactured inner cooling channels highlighted in blue.

In order to optimize the manufacturing process of the mould as much as possible, hybrid production of the tooling was carried out. For this purpose, the WAAM process was performed on the previously machined mould base. As mentioned above, the additive manufacturing process is optimal for complex designs; however, it is not advantageous for the manufacture of the mould base, as it is a flat plate. Therefore, the mould base was manufactured from a steel plate, on which the corresponding areas of the cooling channels were machined, as well as the different forms required (Figure 13). For the manufacturing of this part, a steel block of 50 mm thickness and dimensions of 825 × 1515 mm was used. These dimensions corresponded to the nominal dimensions of the part, to which an extra thickness of 5 mm has was added for subsequent machining.

Moreover, the WAAM process requires a metallic base on which to weld its first layer. Both this plate and the first welded layers are considered as sacrificial and are removed during second-step machining in a standard WAAM manufacturing operation. In our case, we used the mould base plate to start printing, so that we did not waste any material.

**Figure 14:**
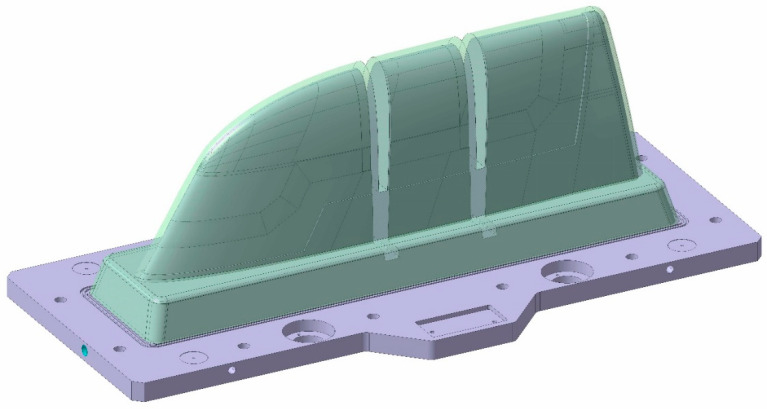
Over-thickness WAAM-process-adapted CAD (green) over final design (grey).

The over-thickness of the WAAM-process-adapted CAD (highlighted in green) is displayed in Figure 14 over the final CAD design. A design over-thickness of 10 mm was chosen for this construction, based on previous tests in the same environment. Considering the manufacturing time and material required, this more conservative over-thickness value was chosen to avoid possible errors due to lack of material during the printing and machining process.

Initially, the selection of the input material for the AM process is crucial. The chosen wire material was G42 4 M21 3Si1 (ISO specification) [81], which is a carbon steel electrode known for its outstanding weldability and high content of deoxidizing elements. This makes it suitable for welding applications where stringent cleaning practices may not be feasible.

Regarding the printing strategy, it followed the following criteria:

- The solid internal area of the mould was printed. This zone is called the filling zone.
- The outer contour of the internal cooling channels was printed.
- The outer contour of the mould was printed.
- The subsequent layer was initiated from a randomly chosen point on the surface to prevent localized heat concentrations in specific areas.

This strategy of printing the infill areas first and the contours afterward allowed us to define the exteriors of the construction. The defined shape of the weld seam in these areas allowed the printing of cantilevered seams. These beads complied with the maximum cantilever angle of 20° [72,73].

**Figure 15:**
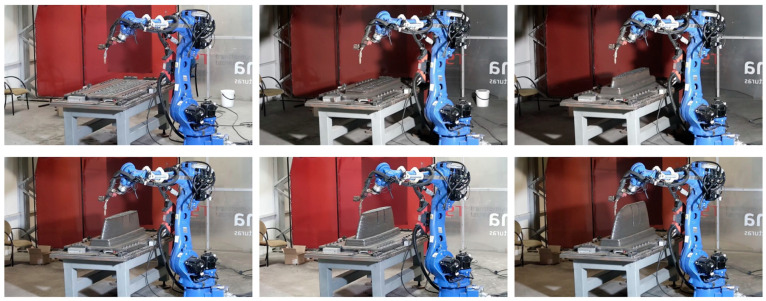
WAAM process manufacturing timelapse.

The WAAM process manufacturing timelapse is shown in Figure 15.

Regarding the WAAM process, its parameters are shown in Table 1.

For the specific case of the WAAM tooling, the total weight of the additive construction was calculated based on the WFR parameter instead of based on the theoretical weight of the over-thickness WAAM-process-adapted CAD fabrication. This parameter shows the actual material consumption, since it includes unexpected material wastes, such as repetitions or printing layer jumps. These anomalies occur when the system sensor detects a growth of the printed piece different from the theoretical one, so the print control loop repeats the printed layer or skips the following one. Another possible anomaly that occurs during the process is the appearance of spatter during welding, which is not reflected in the final weight of the printed tooling but reflected in the consumption of wire.

### 3.3. Cooling Cycle Description

**Figure 16:**
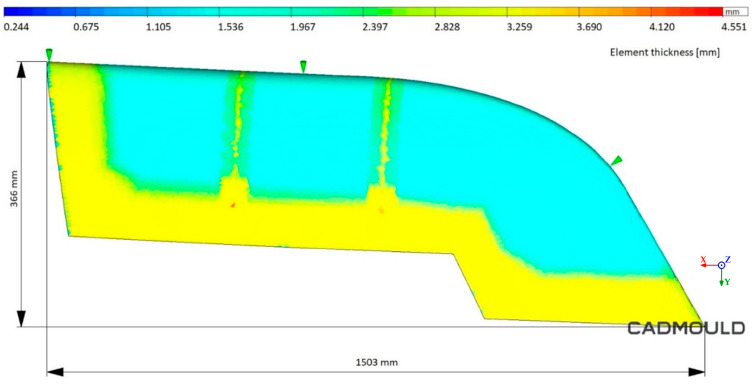
Injected part thickness analysis.

Before the thermal analysis of the process, we must emphasize the final part to be injected. The final geometry, as shown in Figure 16, has a variable thickness between 1.6 and 3 mm. HDPE was the material used for both injection moulding simulations (the WAAM mould and Conventional mould). The injection strategy was a three-point injection. Each injection point is marked as a green arrow in Figure 16.

For the cooling cycle analysis, the following aspects were taken into account:

- The cavity mould, as discussed, is common for both moulds.
- Both moulds have two inserts for part extraction, which have not been taken into account in the previous sections but can be identified in the part as transverse areas of less thickness.
- The simulation was performed with the same parameters for both mould designs, with identical simulation times. The objective is that the results are comparable for both moulds.

**Figure 17:**
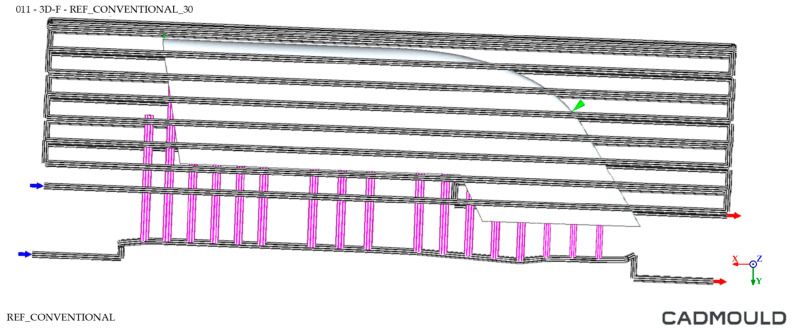
Cooling system simulation environment for Conventional mould.

**Figure 18:**
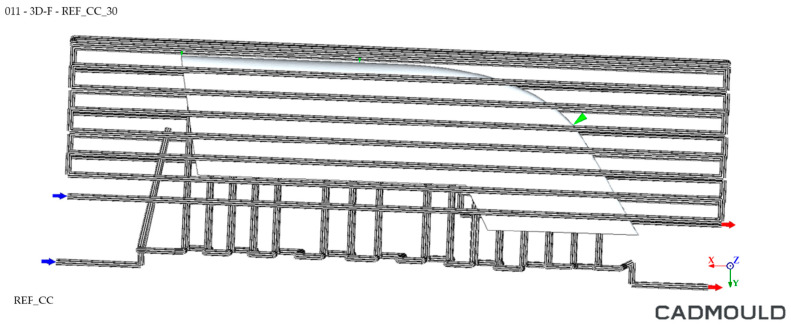
Cooling system simulation environment for WAAM mould.

The cooling system simulation environment comparison for both moulds is shown in Figure 17 and Figure 18, where blue arrows refer to inlet coolant and red arrows the outlet coolant.

Regarding the injection process, the parameters used for the simulation are shown in Table 2. It should be noted that the injected part properties were not only dependent on the cooling process analysis in the study but were also highly dependent on the injection parameters and strategy [82,83,84]. However, by using the same theoretical injection strategy for both simulations, the results were not dependent on these conditions.

One of the most important characteristics of HDPE injection moulding parts is their tendency to shrink during cooling [85]. This process can be controlled and minimized by optimizing the process parameters. The most influential parameters for shrinkage are mould draw temperature and cooling homogeneity. The final part deformation after the cooling cycle was also analyzed.

The following parameters were tested and studied in the analysis:

- The thermal gap in the cooling channels.
- The temperature of the injected part on its inner and outer walls.
- The final deformation of the injected part

## 4. Discussion

### 4.1. Material Consumption Analysis

It should be noted that, as mentioned in the Section 3.2.2, the WAAM mold consists of two parts: base and additive manufacturing material. Due to this fact, material consumption was calculated not considering the material consumptions of each of its parts separately, but the final mould as a whole. Table 3 shows the raw material consumption data for both moulds. Emphasis is placed on the material utilization for both moulds, defined as the percentage of material consumed during the manufacturing process that remains in the final part. The extractable results were as follows:

- The amount of material unloaded for each tooling, shown in terms of mass.
- The percentage of material used in relation to the material consumed.
- The buy-to-fly ratio.

This last concept is defined as the ratio of the weight of raw material used to manufacture the part to the weight of the final part and is used to easily compare material usage in manufacturing parts.

Certain information that is not in Table 3 should be highlighted, such as the total raw material mass required for the manufacturing of each of the toolings. As can be seen, the amount of raw material required for the manufacturing of the theoretical tooling was much higher than that of the WAAM tooling (983.58 kg). As for the final weight of the tooling, calculated using the design software, the weight difference was minimal, with the Conventional mould being 24.58 kg heavier than the WAAM mould. This fact is due to the designed internal channels, which in the case of the WAAM tooling represent a larger volume.

**Figure 19:**
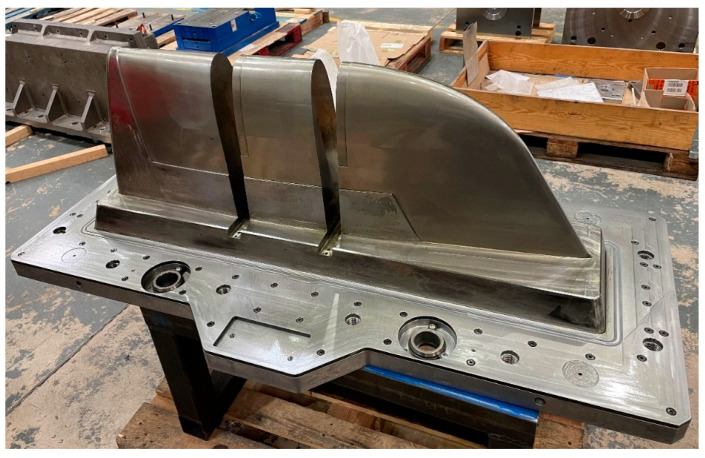
Final WAAM tooling.

The final WAAM mould, once the second-stage machining was completed, is shown in the Figure 19.

The WAAM additive manufacturing process used to manufacture the punch mould should be highlighted. This system makes it possible to manufacture a mould with water-tight internal channels adapted to the geometry of the final part, according to the concept of Conformal Cooling. In addition, it should be noted that in our state-of-the-art review, a mould manufactured in MAM, following the concept of Conformal Cooling design and larger than 300 × 300 × 500 mm, was not found. The tooling presented in this study exceeds these dimensions by a clear margin (820 × 1510 × 512 mm).

### 4.2. Cooling Cycle Analysis

It should be noted that the cooling analysis was made supposing the same initial injection conditions for both moulds, since the cooling cycle was the main feature to be analyzed.

#### 4.2.1. Thermal Gap in Cooling Channels Analysis

**Figure 20:**
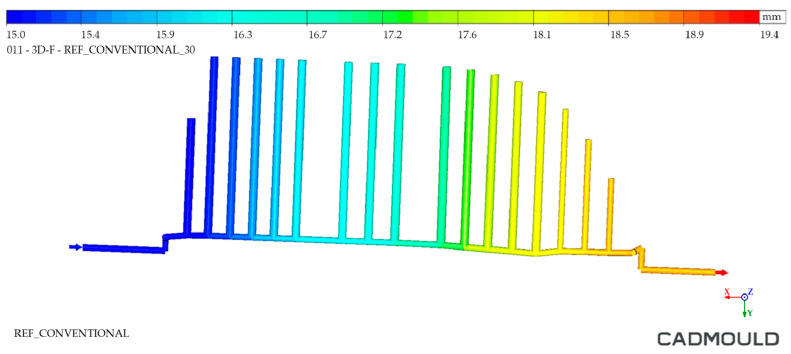
Thermal gap in channels—Conventional mould.

**Figure 21:**
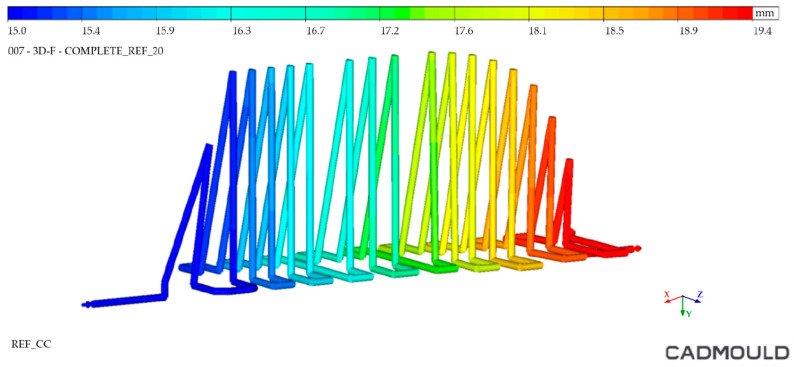
Thermal gap in channels—WAMM mould.

First, the results of the thermal gaps in the cooling channel systems were analyzed. Figure 20 and Figure 21 show the comparison between both cooling channel systems. As can be observed in Figure 20, the thermal jump in the Conventional mould channel system was lower, reaching a thermal difference of 3.7 °C compared to the 4.4 °C thermal difference of the Conformal Cooling channel system (WAAM mould), as displayed in Figure 21. This thermal difference showed that the Conformal Cooling system was able to extract more heat from the part and therefore the cooling water was heated further by 0.7 °C. It should be noted that the thermal jump was high in both cases.

#### 4.2.2. Thermal Distribution on Mould Walls Analysis

**Figure 22:**
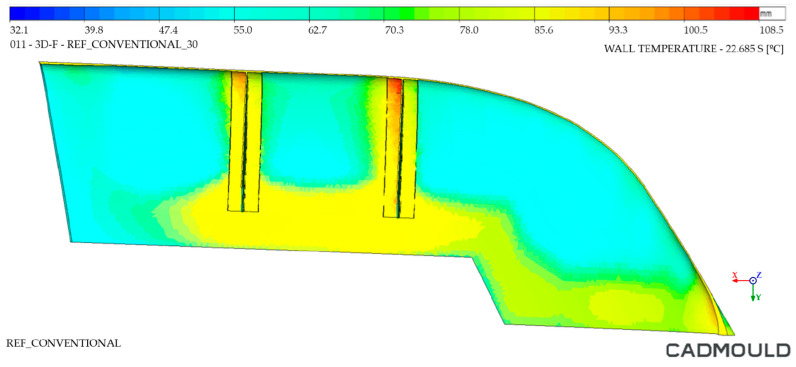
Thermal distribution on inner walls of Conventional mould.

**Figure 23:**
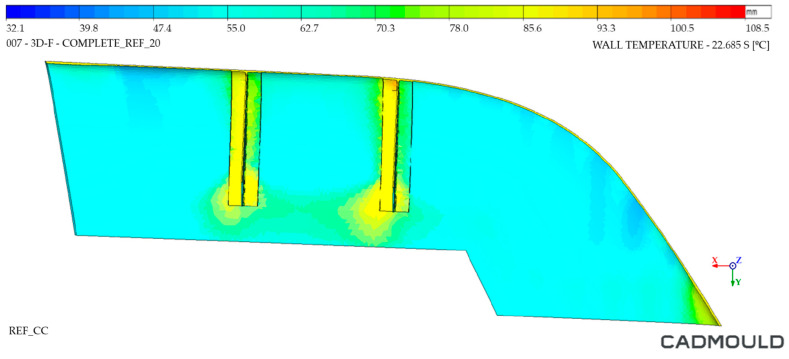
Thermal distribution on inner walls of WAAM mould.

The analysis of the internal and external walls of the injected part after the cooling cycle provided information about the mould surface in these areas. Regarding inner mould walls, Figure 22 and Figure 23 display a temperature comparison for both moulds. As can be seen, the inner wall temperature of the Conventional mould injected part was higher, reaching temperatures up to 87 °C in its most unfavorable zone and 65 °C in its coldest zone. As for the inner wall of the WAAM mould injected part, we observed a maximum temperature of 80 °C and a minimum temperature of 57 °C. When analyzing the temperature distribution, it was observed that the greatest temperature differences were located in the inferior area of the injected part. This is due to the fact that, in the inferior area, the channels of the Conventional mould cooling channels were drilled with a greater distance from the mould walls. In the case of the WAAM mould, its channels’ adaptation to the part geometry provided the benefit of a uniform temperature distribution in the part, in addition to extracting more heat from it.

**Figure 24:**
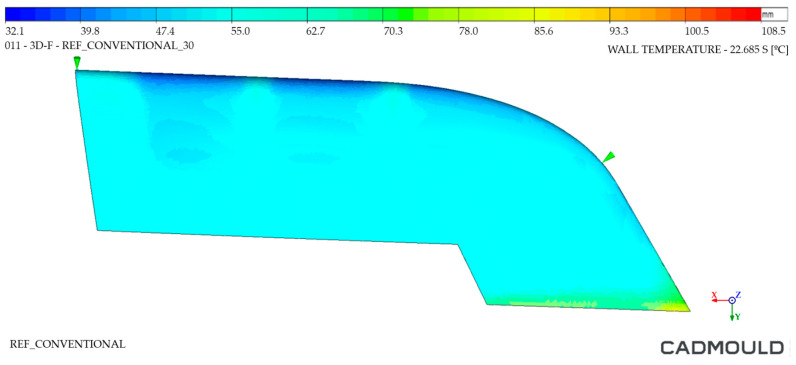
Thermal distribution on outer walls of Conventional mould.

**Figure 25:**
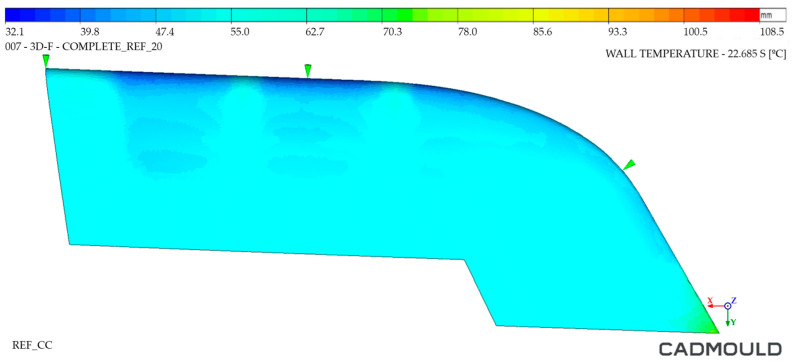
Thermal distribution on outer walls of WAAM mould.

As for the analysis of the outer walls of the part, shown in Figure 24 and Figure 25, there was a slight difference in the lower area of the injected component. Its maximum temperature on the WAAM mould reached 65 °C, 5 °C less than that corresponding to the Conventional mould. This temperature difference was located in the lower zone of the component, also the most unfavorable for the previous analysis. It should be noted that the temperature distribution in the part was quite uniform. In this analysis, a common cavity mould design was used, with the same distribution of cooling channels. For this reason, these thermal differences must be directly related to the heat extraction achieved in the WAAM mould by means of the Conformal Cooling concept.

#### 4.2.3. Injected Part Deformation Analysis

Finally, an analysis of the deformations of the injected part was performed. In this analysis, the deformations that the injected part would undergo when it was removed from the mould at the end of the calculated cooling time (10 s for both moulds in this case) were studied. For an optimal result, a minimum part temperature with uniform distribution across the part should be sought. High draw temperatures lead to higher out-of-mould shrinkage, deforming the injected part. In addition, non-homogeneous temperature distribution in the part can result in surface anomalies such as shininess or marks.

**Figure 26:**
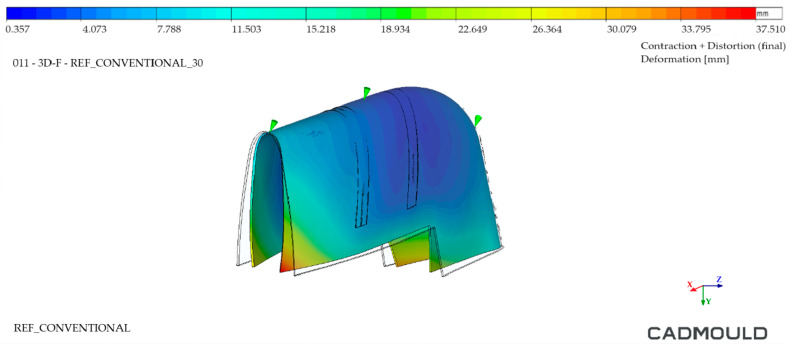
Injected part deformation analysis—Conventional mould.

**Figure 27:**
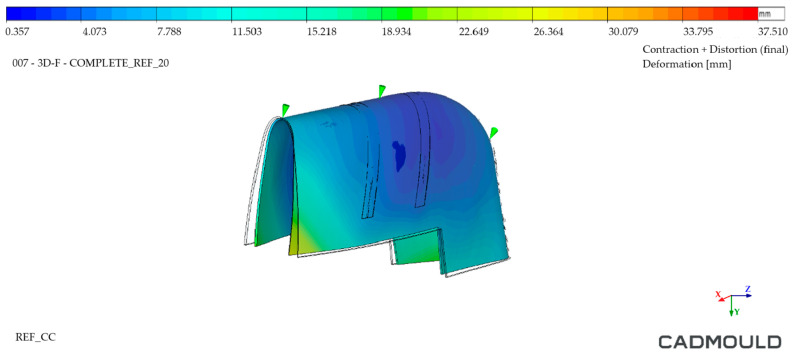
Injected part deformation analysis—WAAM mould.

Figure 26, corresponding to the Conventional mould, displays an important deformation zone in its lower open area. It is worth noting that this is one of the most unfavorable zones for deformation purposes, since HDPE tends to shrink during cooling and these zones have geometric freedom for it, since they do not have internal reinforcements. This same deformation was found in the WAAM mould (Figure 27) but much reduced (35% lower). In addition, it can be seen how the temperature distribution was much more homogeneous in the injected part extracted from the WAAM mould. It should be noted that the deformations at the graphic level have been enlarged in both named figures to facilitate their visualization.

The results obtained through the analysis of the cooling cycle revealed extremely promising improvements in terms of increased homogeneity in the WAAM mould, as well as a significant increase in the amount of heat extracted. This translates into reduced deformation of the final part, thereby reducing distortions to levels comparable to the current state-of-the-art methods. In addition, the higher heat extracted from the WAAM mould can be interpreted as an improvement in the cooling cycle time. It should be noted that the analyses were performed under the assumption of a uniform cooling cycle time for both moulds.

The originality of this study relies on the fact that a real large-dimension mould was manufactured, thanks to WAAM. It was carried out on a physically fabricated large mould, instead of being limited to theoretical simulations. This makes it a relevant case within the state of the art, where previous studies were mainly focused on tools of considerably smaller dimensions or, for the most part, on theoretical tools that have not been produced.

## 5. Conclusions

In conclusion, this article shows the significant suitability of the integration of WAAM technology for the manufacturing of injection moulds. The advantages in terms of material consumption and injection process optimization are evidenced throughout the study.

First of all, the CC-designed WAAM mould was compared with an identical theoretical mould, designed to be produced by conventional manufacturing methods. This conventional mould required a system of baffles as thermal channels, increasing its complexity by requiring auxiliary systems and compromising its watertightness.

A study of the material consumption for the manufacturing of both moulds was carried out. The results showed, for the WAAM mould, a remarkable decrease in the material consumed for manufacturing. The amount of material required for the manufacturing of the WAAM mould was 920.58 kg, which was 4095.44 kg lower than for the conventional mould. Consequently, the percentage of material discarded in the WAAM tooling was 21.56%, and improvement compared to the 84.33% discarded in the conventional mould. This is remarkable because the base plate was a common element in both moulds, with a significant percentage of material utilization. This means the fundamental difference in material savings was that corresponding to the WAAM construction, whose percentage of discarded material was only 9.37%. Finally, the buy-to-fly ratio obtained by the WAAM tooling was only 1.27, compared to the 6.38 of the conventional mould.

Finally, an HDPE injection process simulation and its corresponding thermal analysis for both cooling systems were carried out. As a result, we obtained a greater thermal gap in the inner channel system of the WAAM mould. This data verifies that, for the same period, the CC-based system extracted 0.7 °C more than the system designed in the Conventional mould. This represents an increase of 18.92%. In addition, the analysis of both the outer and lower surfaces of the part showed not only a lower part temperature at the end of the cycle for the WAAM mould, but also a more homogeneous temperature distribution.

In the case of the internal area of the injected part, coincident with the punch mould subject of the study, the difference is more remarkable. The maximum and minimum temperature of this area decreased by 8 °C in the WAAM mould injected part compared to the Conventional mould injected part. Moreover, as the channels of the WAAM mould adapted much better to the geometry of the lower zone of the injected component, the cooling of the part remained homogeneous. In the case of the Conventional mould, in addition to the temperature increase, the influence of the greater distance of the cooling channels from the surface in the lower area of the mould created a high temperature zone.

On the other hand, the results of the analysis of the outer area of the part, corresponding to the mould cavity, show a great similarity. This is because the simulated outer cavity was common for both moulds. However, for the WAAM mould, a lower maximum temperature at the inferior area of the injected part was observed.

As the last element of the study, the deformations of the injected part after the cooling cycle were compared. As in the previous cases, the WAAM mould injected part was found to be superior to the part extracted from the Conventional mould. The deformations of the part injected in the WAAM mould were 26.48% lower in its higher temperature zones. In addition, the temperature distribution was more homogeneous. This homogeneity is desirable to avoid surface defects on the part.

In conclusion, the capabilities of the WAAM process for the manufacturing of large tooling were studied in present research. This study has shown the advantages of the AM process over traditional machining methods in terms of design, manufacturing, and final performance of the manufactured mould. It should be noted that these advantages are situational, depending on the geometry to be manufactured, and should not be taken as a rule when choosing the most appropriate manufacturing method. However, they can serve as a fabrication guideline applicable to future tooling.

## Figures and Tables

**Table 1:** Process parameters for WAAM deposition (courtesy of AITIIP).

Process Parameters	Details	Value
Speed	Welding speed	0.06 m/s
	Wire feed rate	8.64 ± 0.81 m/min
	Deposition rate	0.077 kg/min
Distance and angle	Layer height	1.2 mm
	Electrode to layer angle	90°
Wire	Wire grade	ISO G42 4 M21 3Si1 [81]
	Wire diameter	1.2 mm
	Wire material density	7816.17 kg/m^3^
Shield gas	Shield gas type	ISO 14175-M20-ArC-8 [81] (CO_2_ 8% Ar 92%)
	Shield gas flow rate	15 L/min

**Table 2:** Simulated injection parameters.

Simulated Injection Parameters	
Injection Temperature	245 °C
Injection time	2.6 s
Compaction pressure	400 bar
Compaction time	10 s
Coolant inlet temperature (water)	15 °C
Cooling time (additional to compaction time)	10 s

**Table 3:** Mould weight output comparative.

Mould Punch		Raw Material Weight (kg)	Final Weight (kg)	Discarded Weight (kg)	Discarded Weight (%)	Buy-to-Fly Ratio
WAAM Mould	AM	492.38	446.24	212.05	21.56%	1.27
	Plate	491.20	325.30			
Conventional mould	-	5079.02	796.11	4282.91	84.33%	6.38

## Data Availability

The data supporting the findings of this study are available from the corresponding author upon reasonable request.
